# Unraveling the role of Major Vault Protein as a novel immune-related biomarker that promotes the proliferation and migration in pancreatic adenocarcinoma

**DOI:** 10.3389/fimmu.2024.1399222

**Published:** 2024-07-04

**Authors:** Xinyi Wu, Leiyu Hao, Jianghua Lin, Xinyu Guo, Yuping Luo, Chun Li

**Affiliations:** ^1^ Tongji University Cancer Center, Shanghai Tenth People’s Hospital of Tongji University, Tongji University School of Medicine, Shanghai, China; ^2^ Key Laboratory of Spine and Spinal Cord Injury Repair and Regeneration of Ministry of Education, Orthopedic Department of Tongji Hospital, Tongji University School of Medicine, Shanghai, China

**Keywords:** pancreatic adenocarcinoma, MVP, prognosis, immune-related biomarker, therapeutic target

## Abstract

**Background:**

Pancreatic adenocarcinoma (PAAD) is a formidable challenge in oncology research, with a complex pathogenesis that requires to be explored. Major Vault Protein (MVP) is the principal structural component of the vault complex, and its expression level is remarkably upregulated in various cancers. Extensive investigations have been conducted to explore the role of MVP in specific cancer contexts, yet the potential molecular mechanisms and biological functions of MVP in PAAD still remain considerably elusive. This study aims to explore the role of MVP as a novel immune-related biomarker in the pathogenesis and clinical treatment of PAAD.

**Methods:**

Gene expression data and clinical information were collected from TCGA, GTEx and GEO databases. Survival, prognostic and functional enrichment analysis were employed with R software. Immunological correlation analysis was performed using TIMER2.0, TIDE scores, TISIDB and TISCH. Epigenetic analysis was implemented by MethSurv, CPTAC, UALCAN, and cBioPortal. Drug analysis was conducted using Enrichr and CellMiner. Moreover, cellular experiments, like RNA interference, qRT-PCR, Western blot, cell cycle analysis, cell apoptosis analysis, colony formation assay, transwell assay, and wound healing assay, were performed for verifying the functional properties of MVP in the PAAD progression.

**Results:**

We demonstrated an abnormally upregulated expression of MVP in PAAD tissues, which notably correlated with an adverse prognosis in PAAD patients. Functional analysis suggested the conceivable involvement of MVP in immune modulation, and immunotherapy. Additionally, we identified genetic alterations, reduced promoter methylation, and heightened phosphorylation in MVP. We also clarified Suloctidil and Tetradioxin as the most notable potential drugs targeting MVP in PAAD. Moreover, our experimental observations consistently highlighted the significant impact of MVP deficiency on impeding PAAD cell proliferation, inhibiting cell migration, and accelerating cell apoptosis. Interestingly, a potential link between MVP and ERK or AKT pathways was displayed, which opens new avenues for further exploration of the molecular mechanisms of MVP-targeted therapies in PAAD.

**Conclusions:**

This study systematically describes MVP as an immune-related biomarker with remarkable potential for predicting the prognosis, tumor progression and immunotherapeutic efficacy in PAAD.

## 1 Introduction

Pancreatic adenocarcinoma (PAAD) poses a formidable challenge in the field of oncology due to its aggressive nature and limited therapeutic options, with a disheartening 5-year survival rate of approximately 10% as of 2020 (1). The advancements in the landscape of molecular profiling for PAAD has steadily improved the management of this malignancy over the past decade (2). Tumor markers like CA19–9, CA125, and CEA have been used for routine diagnosis of PAAD (3). Emerging insights suggest the diagnostic potential of CD1D methylation levels in pancreatic juice, presenting an impressive AUC value (up to 0.92), with 75% sensitivity and 95% specificity (4). In addition, elevated SNHG15 levels in PAAD patients, which promote cell proliferation through EZH2-mediated H3K27me3, also provide new perspectives for PAAD diagnosis (5).

Despite these advancements, the complexity of molecular mechanisms underlying the pathogenesis of PAAD remains an enigma, a more comprehensive and in-depth analysis of the molecular regulatory networks is urgently needed to pave the way for more effective therapeutic strategies. Among the molecular participants associated with tumorigenesis, Major Vault Protein (MVP) has become a research focus due to its role as a major component of multi-subunit ribonucleoprotein particles (known as vaults) which involved in nuclear-cytoplasmic transport. MVP was first isolated and characterized from rat liver coated vesicles in 1986 (6). In 1995, a study found that MVP, also known as the drug resistance-related protein (LRP), is the human major vault protein, and that its overexpression leads to a poor response to chemotherapy in acute myeloid leukemia and ovarian carcinoma (7). Subsequent research has further elucidated the multiple roles of MVP in cancer biology. For instance, it has been identified as a promoter in the development of papillary thyroid cancer, serving as an immune microenvironment-associated biomarker (8). In glioblastoma, MVP enhances temozolomide-resistance, and is considered as a novel biomarker for glioblastoma stem cells (9). In colon cancer, elevated MVP expression not only promotes metastasis of colon cancer cells but also confers resistance to vinorelbine, revealing its multifaceted impacts on cancer progression (10). A recent study has provided compelling evidence for the role of MVP in hepatocellular carcinoma (HCC). The researchers found that MVP promoted HCC proliferation and metastasis by regulating M2 polarization both in *in vivo* and *in vitro*, suggesting the potential involvement of MVP in modulating the tumor-associated macrophages (TAMs) within the tumor microenvironment (TME) (11). These discoveries illuminate that MVP has made diverse and meaningful contributions to oncology research, underscoring its crucial role in the intricate network of molecular events that govern cancer progression. However, the functional significance of MVP has yet to be elucidated in PAAD. Hence, we sought to comprehensively explore the potential role of MVP in the occurrence, progression and clinical treatment of PAAD.

Through a comprehensive integration of bioinformatics analysis and experimental investigations, our study unveils that MVP serves not only as a novel biomarker that accelerates the malignant progression of PAAD, but also exerts a noticeable influence on the TME in PAAD. In conclusion, these results recapitulate the important contribution of MVP to the intricate landscape of PAAD, shedding light on its potential as an immune-related biomarker.

## 2 Materials and methods

### 2.1 Data acquisition

Gene expression data and associated clinical data for PAAD patients were downloaded from The Cancer Genome Atlas (TCGA, https://portal.gdc.cancer.gov/) database and Gene Expression Omnibus (GEO, https://www.ncbi.nlm.nih.gov/geo/) database (GSE15471, GSE62165, GSE16515 and GSE62452). Additional normal sample data were obtained from Genotype-Tissue Expression (GTEx). We utilized the Gene Expression Profiling Interactive Analysis 2.0 (GEPIA2) platform (http://gepia2.cancer-pku.cn/) to investigate the expression patterns of MVP across multiple cancer types.

### 2.2 The tissue microarray (TMA)

The TMA was purchased from Shanghai Outdo Biotech, including 10 primary PAAD tumor tissues and 10 matched adjacent normal tissues for the analysis of MVP expression patterns.

### 2.3 Survival analysis and prognostic analysis

Based on the TCGA-Clinical Data Resource, the correlation between the expression of MVP and the survival rate for different clinical characteristics was estimated using the survival package and the survminer package. The survival analyses were assessed by the Kaplan-Meier curves and Cox proportional hazard regression models. The TCGAplot package (12) was utilized to plot receiver operating characteristic (ROC) curve.

### 2.4 Gene functional enrichment analysis

A heatmap of the most differentially expressed genes based on MVP expression and gene set enrichment analysis (GSEA) was generated by TCGAplot (12). Gene Ontology (GO) and Kyoto Encyclopedia of Gene and Genomes (KEGG) pathway analysis were conducted to analyze the biological processes and signal pathways in which genes with the strongest association with MVP in PAAD may participate. STRING (https://cn.string-db.org/) and GeneMANIA (https://genemania.org/) were utilized to analyze the protein–protein interaction network.

### 2.5 Immunological correlation analysis

Correlation data between the expression of MVP and 28 kinds of tumor-infiltrating immune cells (TIICs) were downloaded from the TIMER2.0 (http://timer.cistrome.org/) (13). The Tumor Immune Dysfunction and Exclusion (TIDE) scores and Microsatellite Instability (MSI) status of PAAD samples (GSE15471 and GSE62542) were calculated by the TIDE database (http://tide.dfci.harvard.edu/). TISIDB database (http://cis.hku.hk/TISIDB/) was applied to investigate the association between MVP expression and immunomodulators (14). Furthermore, TISCH database (http://tisch.comp-genomics.org/home/) (15) was performed to investigate the relationship between MVP expression levels and immune cell at the single-cell level.

### 2.6 Methylation and phosphorylation analysis

The cluster analysis of individual CpGs of MVP in PAAD patients and the assessment of survival differences based on methylation levels at different CpG sites of MVP in patients were conducted using MethSurv (https://biit.cs.ut.ee/methsurv/) (16). Differential analysis of MVP phosphorylation levels and protein abundance between PAAD and adjacent tissues were performed using the cProSite function in CPTAC (https://pdc.cancer.gov/pdc/). The promoter methylation levels of MVP in PAAD were analyzed by UALCAN (https://ualcan.path.uab.edu/index.html). Additionally, the alternation frequency of MVP across various cancer types was calculated from the cBioPortal (https://www.cbioportal.org/) based on the study of Pan-cancer analysis of whole genomes (ICGC/TCGA).

### 2.7 Analysis of drug treatment and potential drug forecasting

The CellMiner (https://discover.nci.nih.gov/cellminer/home.do) online database was utilized to investigate the correlation between drug treatment effects and gene expression levels (17). Additionally, with the Enrichr’s Drug Signature Database (https://maayanlab.cloud/Enrichr/), we predicted potential drug candidates targeting MVP and ranked these candidates based on their p-values, with lower p-values indicating higher priority. The structure of these predicted drugs was downloaded from Pubchem (https://pubchem.ncbi.nlm.nih.gov/).

### 2.8 Cell culture and RNA interference

Miapaca-2 and sw1990 cells obtained from ATCC were cultured in DMEM medium (WISENT, 319–005-CL) supplemented with 10% FBS and 1% Pen/Strep in a 5% CO2 incubator at 37°C. These two cell lines were transfected with lipofectamine 2000 (Thermo Fisher Scientifc, Inc.) mixed with siRNA to knock down the expression of MVP, respectively. The antisense sequence of the siRNA (siMVP) is: 5’- CCUACAUGCUGACCCAGGATT-3’.

### 2.9 qRT−PCR

Total RNA was isolated using RNA isolater Total RNA Extraction Reagent (Vazyme, China) following the manufacturer’s protocol. cDNA was synthesized using the PrimeScript RT reagent Kit with gDNA Eraser kit (Takara, Japan). Quantitative PCR (qPCR) was carried out using ChamQ Universal SYBR qPCR Master Mix (Vazyme, China). The specific primer sequences utilized are provided below.

MVP-F: 5’-CAACTACTGCGTGATTCTC-3’; MVP-R: 5’- TCAGCATGTAGGTGCTTC-3’
ACTIN-F: 5’-CATGTACGTTGCTATCCAGGC-3’; ACTIN-R: 5’-CTCCTTAATGTCACGCACGAT-3’

### 2.10 CCK8 assay

24 hours after transfection, PAAD cells (3 × 10^3^ cells per well) were inoculated in 96-well plates and cell viability was estimated with CCK8 (Beyotime, China) at 24-, 48- and 72-hours post-transfection, respectively.

### 2.11 Cell cycle analysis

After a 72-hour transfection with siMVP or siNC, miapaca-2 and sw1990 cells were subjected to flow cytometry analysis to assess the impact of MVP knockdown on the cell cycle. The cells were trypsinized, centrifuged at 1200 rpm for 5 minutes, and washed three times with pre-chilled PBS. The cells were fixed with 70% ethanol at -20°C overnight and then the cell cycle distribution was determined using a cell cycle kit (Beyotime, China) and a flow cytometry (BECKMAN COULTER, USA).

### 2.12 Western blot

Cells were collected for protein extraction with RIPA lysis buffer (Beyotime, China). Western blot was performed as previously reported (18) The following primary antibodies were used: anti-MVP (proteintech, China), anti-GAPDH, anti-Caspase-3, anti-Survivin, anti-AKT, anti-Phospho-Akt, anti-p44/42 MAPK (Erk1/2), anti-Phospho-p44/42 MAPK (Erk1/2) were purchased from Cell Signaling Technology.

### 2.13 Colony formation assay

Transfected PAAD cells were seeded into 6-well plates at a density of 1000 cells/well for 7 days. The colonies were then fixed with 4% polyformaldehyde for 15 minutes and then stained with crystal violet for 30 minutes. The number of colonies was evaluated by ImageJ software.

### 2.14 Wound healing assay

PAAD cell lines (Miapaca-2 and sw1990) were seeded into 6-well plates, respectively. When the cell confluency reached almost 80%, the monolayer cells were scratched into a straight line with a sterile micropipette tip. The wound healing within the scrape line was observed at 0- and -24 hours. Corresponding images were captured and subsequently analyzed using ImageJ software.

### 2.15 Transwell assay

Transwell assay was conducted to assess cell migration capacity. After transfected with MVP siRNA, PAAD cells were harvested and seeded at a density of 1 × 10^5^ cells/well in medium containing 200 μL of serum-free medium above an 8 μm chamber (14341, LABSELECT, China). 500 μL of medium containing 10% FBS was added to lower chamber. After 24 hours of incubation, cells that had migrated into the lower chamber were fixed with 4% polyformaldehyde for 15 minutes and subsequently stained with crystal violet for 30 minutes. The number of stained cells was quantified by ImageJ software.

### 2.16 Cell apoptosis analysis

The Annexin V-FITC/PI Apoptosis Detection Kit (CX006S) was purchased from Epizyme Biomedical. Adherent cells and suspension cells in culture medium were collected 72 hours after transfection, cells were rinsed with PBS, centrifuged, resuspended in 100 μl binding buffer, and then 5 µl of Annexin V-FITC and 10 µl of PI were added. The mixture was incubated for 15 minutes under dark conditions. Flow cytometry (BD FACSCanto™II system, USA) was utilized for detecting the proportion of apoptotic cells.

### 2.17 Cell viability assay

Transfected PAAD cells were seeded in 96-well plates at a density of 3 × 10^3^ cells/well. Following overnight incubation, cells were treated with varying concentrations of Gemcitabine (a chemotherapy agent widely used in the clinical treatment of PAAD) ranging from 0.004 μM to 3 μM in threefold increments for 72 hours. The cell viability was detected as the protocol provided by manufacturer for CellTilter Glo Luminescent Cell Viability Assay (Promega).

### 2.18 Statistical analysis

All statistical analyses were visualized via GraphPad Prism 9.0 or R software (version 3.1.6 and 4.3.2). The established R packages were utilized as mentioned above. In addition, various statistical tests, including the chi-squared test, Student’s t-test, two-way ANOVA, Wilcoxon test, and Kruskal–Wallis test, were employed to determine the significance of observed differences between distinct groups. Statistical significance is described as follows: ns, not significant; *, P < 0.05; **, P < 0.01; ***, P < 0.001; ****, P < 0.0001.

## 3 Results

### 3.1 The abnormally elevated expression of MVP in PAAD

**Figure 1:**
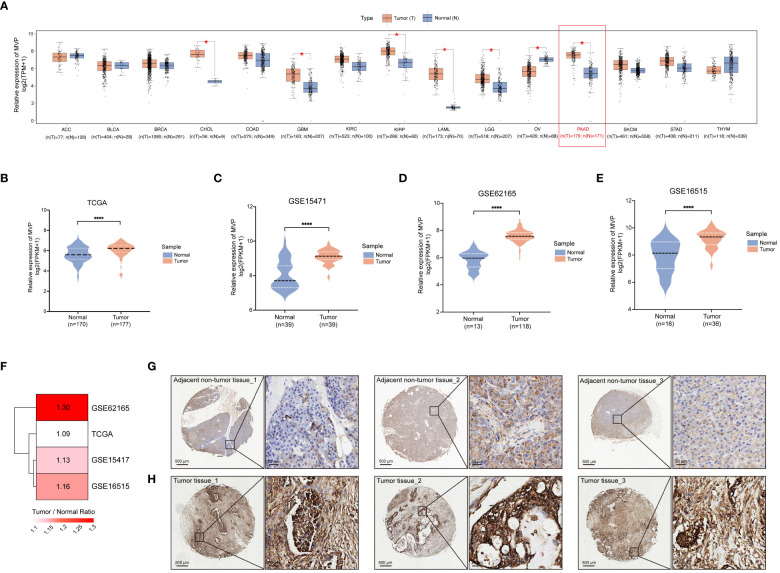
Increased expression level of MVP is shown in PAAD compared to normal tissues. **(A)** The expression level of MVP is elevated in PAAD compared to normal tissues from the GEPIA 2.0. Gene expression data are normalized as log2 (TPM+1). TPM: Transcripts Per Million **(B–E)** The expression of MVP in TCGA (N=170, T=177), GSE15471 (N=39, T=39), GSE62165 (N=13, T=118), and GSE16515 (N=16, T=36) datasets. Gene expression data are normalized as log2 (FPKM+1). FPKM: Fragments Per Kilobase of transcript per Million mapped reads **(F)** The heatmap illustrating the degree of upregulated expression (Tumor/Normal ratio) of the MVP gene across different datasets of PAAD. **(G, H)** Representative immunohistochemistry images of MVP expression in PAAD tumor tissues and adjacent non-tumor tissues. Scar bars, 500 μm and 20 μm. Statistical differences were assessed by using unpaired Student´s t-test (*p < 0.05; ****p < 0.0001).

Genes exhibiting dysregulated expression in tumor samples are considered to be potentially involved in tumor occurrence and progression. Thus, we analyzed the expression of MVP across 15 diverse human cancers from the TCGA database through GEPIA2, and found that the expression of MVP was significantly increased in PAAD compared to normal tissues (**Figures 1A, B**). This aberrant expression pattern was further validated in the GSE15471, GSE62165 and GSE16515 of GEO database (**Figures 1C–F**). Subsequently, to corroborate these observations, we examined the protein levels of MVP in 10 primary PAAD tumor tissues and 10 matched adjacent normal tissues. As expected, immunohistochemistry (IHC) analysis further confirmed that MVP was expressed at higher levels in PAAD than in normal tissues (**Figures 1G, H**).

### 3.2 Clinicopathological correlation, prognostic and diagnostic value of MVP in PAAD

In order to comprehensively assess the clinical significance of MVP, we evaluated the correlation between distinct clinical parameters (race, tumor grade, pancreatitis status, and TP53 mutation status) of PAAD and the expression levels of MVP using the UALCAN database. The results revealed the notably elevated expression of MVP in Grade 3 tumors compared to other grades, the obviously higher expression in patients with pancreatitis than those without pancreatitis or normal individuals, and the significantly upregulated expression in patients with TP53-mutant PAAD compared to those with the non-mutant type (**Supplementary Figures 1A–D**). Previous research has shown a large proportion of TP53 mutations (64%-83%) in PAAD cases (19), which is consistent with our findings, thus suggesting the potential of MVP as a promising biomarker in clinical diagnosis of PAAD.

**Figure 2:**
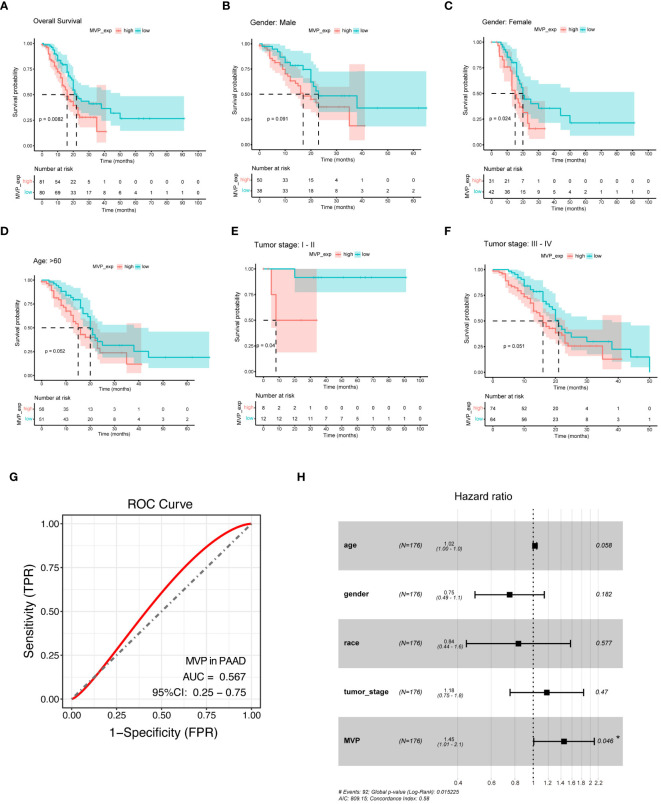
MVP is a promising diagnostic and prognostic indicator in PAAD. **(A)** Kaplan-Meier (KM) survival curve analysis of overall survival (OS) contrasts MVP high- and low- expression groups in PAAD patients from TCGA. **(B–F)** Analysis of KM survival curves in male, female, age>60, tumor stage I-II and tumor stage III-IV of PAAD patients. **(G)** ROC curve of MVP in PAAD (AUC=0.567). **(H)** Multivariate cox regression analysis of MVP in TCGA.

To evaluate the prognostic value of MVP in PAAD, we stratified the TCGA-PAAD cohort into MVP high- and low- expression groups, and performed Kaplan-Meier (KM) survival curve analysis of overall survival (OS), which revealed that the elevated expression of MVP was a prognostic indicator associated with a markedly poor prognosis. (**Figure 2A**). Notably, this prognostic value remained consistent across specific clinical parameters, including gender, age and tumor stage (**Figures 2B–F**; **Supplementary Figures 1E–H**). In addition, the receiver operating characteristic (ROC) analyses were employed to assess the diagnostic value of MVP, and the area under the curve (AUC) was 0.567, indicating its potential as a diagnostic indicator for PAAD (**Figure 2G**). We further explored the MVP-related OS via multivariate Cox regression analyses, which confirmed the high MVP expression as a significant risk factor for PAAD (**Figure 2H**).

### 3.3 Functional enrichment analysis of MVP-related genes in PAAD

**Figure 3:**
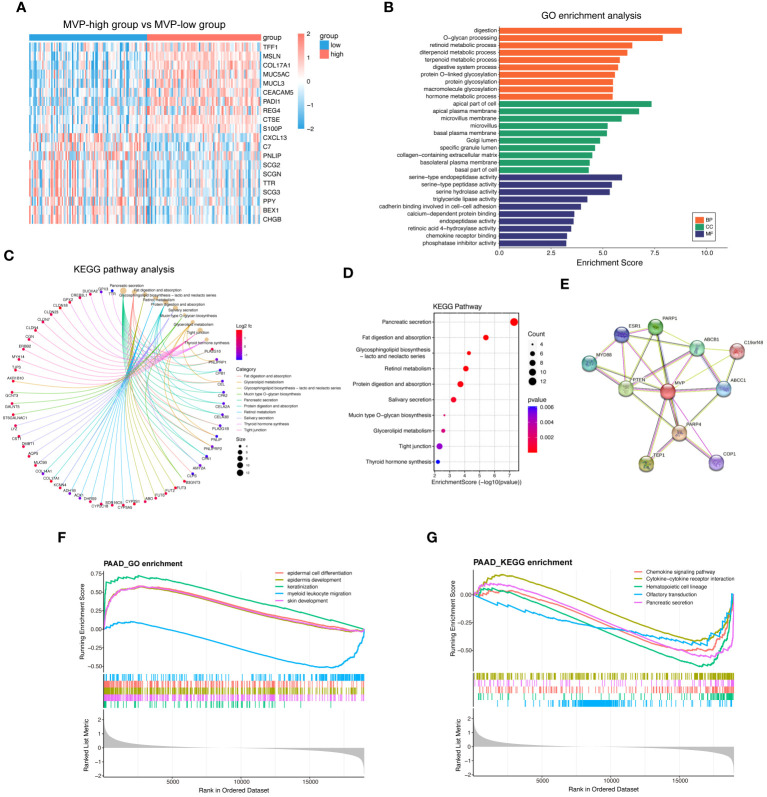
Functional enrichment analysis of MVP-related genes in PAAD. **(A)** Top 20 differential genes in MVP high- and low- expression groups from TCGA-PAAD cohort. **(B)** GO enrichment analysis of differential genes. **(C, D)** KEGG pathway analysis of differential genes. **(E)** PPI network of MVP. **(F)** GSEA_GO enrichment of MVP in PAAD. **(G)** GSEA_KEGG enrichment of MVP in PAAD.

To elucidate the genes correlated with MVP and to further explore the biological functions of MVP in the development of PAAD, we performed functional enrichment analysis of the differential gene expression profiles between MVP high- and low- expression groups. The volcano plot displays the landscape of differentially expressed genes (DEGs) between these groups (**Supplementary Figure 2A**), and the heatmap exhibits the top 10 up- and down-regulated DEGs (**Figure 3A**). After the screening of GO enrichment analysis with those DEGs related to MVP, we focused on the top 30 GO terms containing biological process (BP), cellular component (CC) and molecular function (MF). BP is mainly enriched in “digestion” and “O-glycan processing”; CC is primarily related with “apical part of cell” and “apical plasma membrane”; MF shows a relatively high relationship with “serine-type endopeptidase activity” and “serine-type peptidase activity” (**Figure 3B**; **Supplementary Figures 2B–D**). KEGG analysis indicates that “pancreatic secretion”, “fat digestion and absorption”, and “glycosphingolipid biosynthesis” might be involved in the role of MVP in tumor pathogenesis of PAAD (**Figures 3C, D**; **Supplementary Figure 2E**). To explore the proteins that potentially interact with MVP, we constructed a protein-protein interaction (PPI) network with STRING database and geneMANIA database, which revealed potential interactions between multiple proteins and MVP, such as PTEN, PARP1, and PARP4 (**Figure 3E**; **Supplementary Figure 2F**).

Additionally, we utilized Gene Set Enrichment Analysis (GSEA) to elucidate biological functions and signaling pathways in which MVP may be involved in PAAD. GSEA indicated that the most enriched GO terms were associated with “epidermal cell differentiation” and “epidermis development” (**Figure 3F**), and that the enrichment in KEGG pathways were primarily related to the “chemokine signaling pathway” and “cytokine-cytokine receptor interaction” (**Figure 3G**), suggesting potential functions of MVP in immunomodulation and related signaling events.

### 3.4 Evaluation of the immunomodulatory and immunotherapeutic roles of MVP in PAAD

As we have known that immune regulation plays a key role in tumor initiation, growth, and treatment, herein, we carried out a multifaceted investigation to assess the relationship between MVP and immunity.

**Figure 4:**
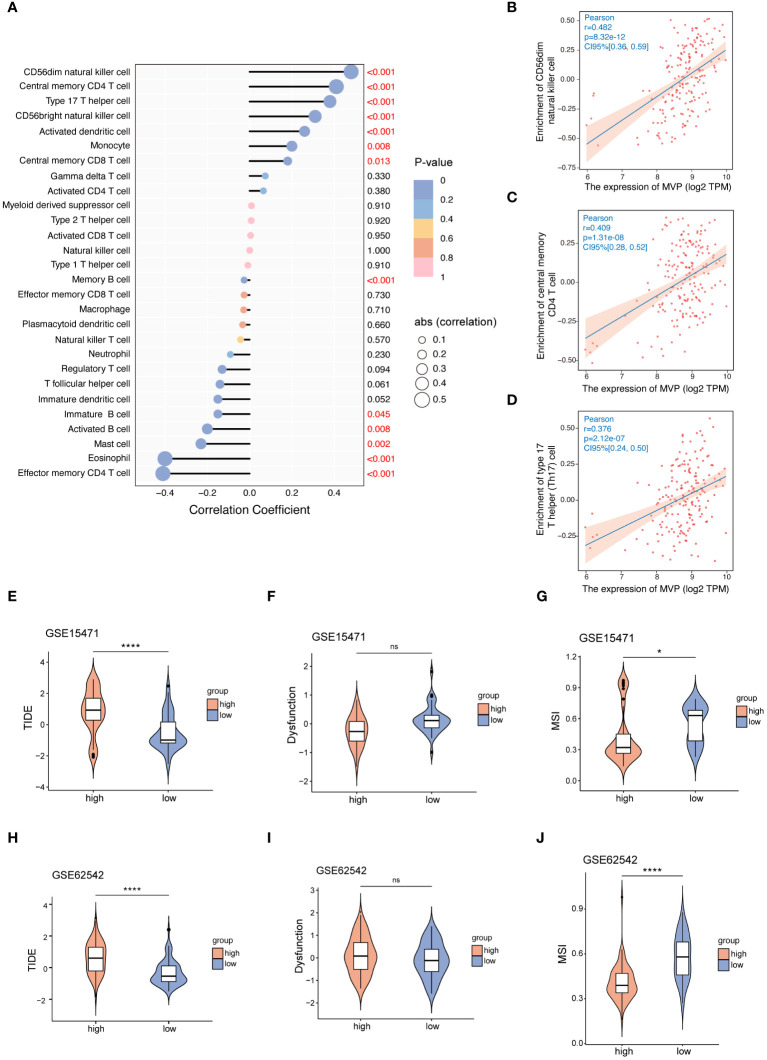
The correlation between the expression of MVP and TIICs or immune checkpoint therapy. **(A)** The correlation coefficient between MVP expression and TIICs in PAAD. **(B–D)** The three TIICs with the strongest positive correlation to MVP expression in PAAD. Gene expression data are normalized as log2 TPM. **(E–J)** The TIDE, Dysfunction and MSI scores of MVP high- and low- groups in GSE15471 and GSE62542 datasets. * p < 0.05; **** p < 0.0001; ns, not significant.

Firstly, the GSEA_KEGG enrichment analysis suggests that MVP is associated with immune regulation (**Figure 3G**). Then we clarified the correlation between the expression of MVP and various kinds of tumor-infiltrating immune cells (TIICs) in PAAD. We downloaded the correlation data between the expression of MVP and 28 kinds of TIICs in PAAD via Timer2.0, and the lollipop plot depicted a predominantly significant correlations between MVP and a cohort of TIICs, particularly prominent for CD56dim natural killer cell, central memory CD4 T cell and Type 17 T helper (Th17) cell (**Figures 4A–D**).

Subsequently, we sought to elucidate the relationship between MVP expression and ICI (immune checkpoint inhibitor) therapy, an emerging paradigm in pancreatic carcinoma treatment (20). Tumor immune dysfunction and exclusion (TIDE), a computational method that mimics the two major mechanisms of tumor immune evasion, has the ability to predict the prognosis of patients with tumors receiving first-line anti-PD1 or anti-CTLA4 therapy (21). It is commonly assumed that elevated TIDE scores and dysfunction scores are correlated with an increased probability of immune evasion, suggesting diminished efficacy of ICI therapy for patients. Contrarily, microsatellite instability (MSI) scores exhibit an inverse relationship. Therefore, we accessed TIDE scores, dysfunction scores and MSI scores of GSE15471 and GSE62542 datasets (**Figures 4E–J**). The results revealed an increased tendency for better benefit from ICI therapy with decreased expression level of MVP, which is consistent with the better prognosis observed in the MVP low expression group shown in **Figure 2**.

### 3.5 Exploration of the interaction between MVP and immune modulators in PAAD

**Figure 5:**
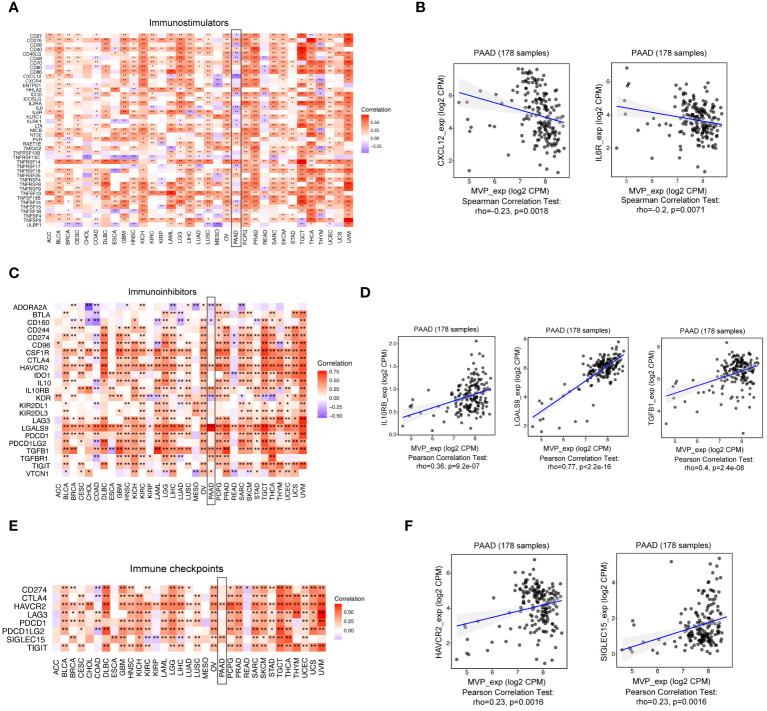
The correlation between MVP expression levels in PAAD and key immune-related factors. **(A, B)** The correlation between MVP expression and immunostimulators in TCGA-PAAD. Gene expression data are normalized as log2 CPM. CPM, Counts Per Million **(C, D)** The correlation between MVP expression and immunoinhibitors in TCGA-PAAD. Gene expression data are normalized as log2 CPM. **(E, F)** The correlation between MVP expression and immune checkpoints in TCGA-PAAD. Gene expression data are normalized as log2 CPM. * p < 0.05; ** p < 0.01.

Immunomodulators and chemokines play momentous roles within the intricate landscape of the tumor microenvironment (TME), promoting tumor development and metastasis (22). To investigate the correlation between MVP expression levels in PAAD and key immune-related factors, we analyzed data from the TISIDB website. Our findings demonstrate a predominantly negative correlation between MVP expression and immunostimulatory factors (**Figures 5A, B**), yet positive correlations with immunoinhibitors (**Figures 5C, D**), immune checkpoints (**Figures 5E, F**), chemokines (**Supplementary Figures 3A, B**), and chemokine receptors (**Supplementary Figures 3C, D**).

### 3.6 The expression profile of MVP across distinct immune cell types in PAAD at single-cell resolution

**Figure 6:**
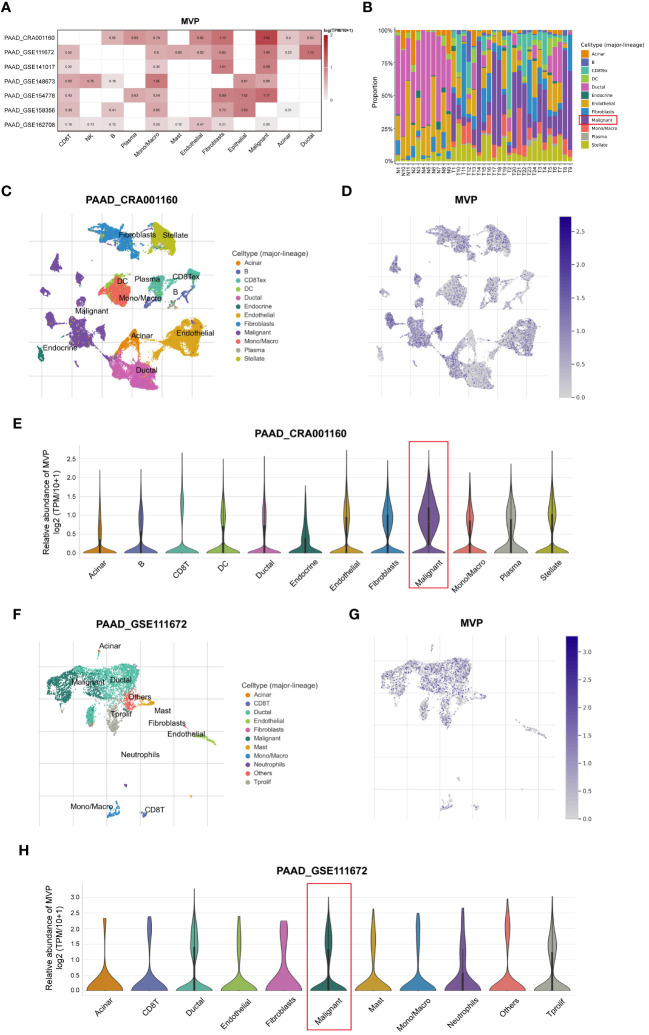
Analysis of the MVP expression pattern in different immune cells of PAAD at single-cell resolution. **(A)** The correlation between MVP and immune cells in PAAD based on TISCH database. **(B)** The proportions of various immune cell populations in PAAD patients compared to normal tissues. **(C)** UMAP plot of immune cells within tumor tissues in PAAD_CRA001160. **(D)** UMAP plot of MVP expression within tumor tissues in PAAD_CRA001160. **(E)** The relative abundance of MVP in various immune cells in PAAD_CRA001160. Gene expression data are normalized as log2 (TPM/10 + 1). **(F)** UMAP plot of immune cells within tumor tissues in PAAD_GSE111672. **(G)** UMAP plot of MVP expression within tumor tissues in PAAD_GSE111672. **(H)** The relative abundance of MVP in various immune cells in PAAD_GSE111672. Gene expression data are normalized as log2 (TPM/10 + 1).

To explore the correlation of immune cell distribution with MVP expression levels at the single-cell level, we obtained 7 independent datasets of PAAD (PAAD_CRA001160, PAAD_GSE111672, PAAD_GSE141017, PAAD_GSE148673, PAAD_GSE154778, PAAD_GSE158356, and PAAD_GSE162708) from the scRNA-seq TISCH database (**Figure 6A**). The proportion of malignant cells in PAAD tumor tissues exceeded that in normal tissues, as illustrated in **Figure 6B**. Moreover, we examined the PAAD_CRA001160 and PAAD_GSE111672 cohorts for deeper investigation. In the PAAD_CRA001160 cohort, a UMAP plot of immune cells within tumor tissues (**Figure 6C**) revealed a widespread distribution of MVP among various immune cell types (**Figure 6D**), with the highest percentage observed in malignant cells (**Figure 6E**). This observation was consistent in the PAAD_GSE111672 cohort, as depicted in **Figures 6F–H**.

Taken together, these results suggest that MVP may promote tumorigenesis by modulating the immune system, and that PAAD patients with heightened MVP expression may be less responsive to immunotherapeutic response such as ICI therapy.

### 3.7 Genetic alterations, promoter methylation and phosphorylation analysis of MVP in PAAD

**Figure 7:**
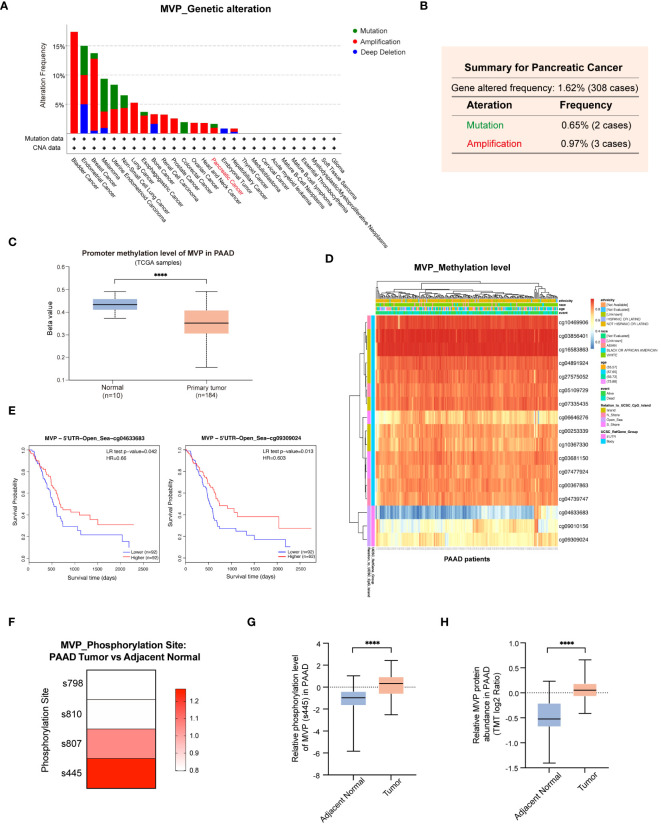
Genetic alterations, promoter methylation and phosphorylation analysis of MVP in PAAD. **(A)** Genetic alterations of MVP in pan-cancer. **(B)** Genetic alterations of MVP in PAAD (mutation=0.65%, amplification=0.97%). **(C)** The promoter methylation level of MVP in PAAD compared to normal tissues. **(D)** The heatmap of MVP methylation level at 17 sites in PAAD. **(E)** The KM curves of MVP related survival analysis with the methylation sites cg04633683 and cg09309024. **(F)** Phosphorylation mutation site of MVP in PAAD compared to normal tissues. **(G)** The phosphorylation level of MVP (s445) in PAAD compared to normal tissues. **(H)** The relative MVP protein abundance in PAAD compared to normal tissues. The TMT (Tandem mass tagging) log2 ratio is a logarithmically transformed measure that quantifies the relative protein expression levels, enabling precise analysis and comparison within TMT-based experimental setups. ****p < 0.0001.

To comprehensively unravel the aberrant alterations of MVP in PAAD initiation and progression, we conducted an in-depth investigation of the genetic alterations, promoter methylation level, and phosphorylation status of MVP in PAAD. The landscape of genetic alterations of MVP in pan-cancer were detected via the cBioPortal database, which unveiling a prevalence pattern of genetic variation in MVP within the cancer spectrum (**Figure 7A**). Generally, the main type of its genetic alterations was “amplification”, “mutation”, and “deep deletion”. In the case of PAAD, we analyzed 308 PAAD patients with a frequency of MVP gene alterations up to 1.62%, containing a “mutation” rate of 0.65% and an “amplification” rate of 0.97% (**Figure 7B**).

Upon scrutiny of the UALCAN database, we found a marked reduction in the promoter methylation level of MVP in primary tumors compared to normal tissues (**Figure 7C**). To further investigate the methylation pattern of MVP in PAAD, we utilized the MethSurv database and identified 17 methylation sites that have a significant impact on MVP expression (**Figure 7D**). Notably, the methylation levels of most sites were positively correlated with survival, such as cg04633683 and cg09309024, whose KM analysis demonstrated consistency with the MVP related survival analysis in PAAD patients (**Figure 7E**).

Moreover, we explored phosphorylation mutations of MVP in PAAD using the Clinical Proteomic Tumor Analysis Consortium (CPTAC) database. Our research findings indicated that in PAAD, phosphorylation mutation sites of the MVP protein were located at the s807 and s445 sites (**Figure 7F**), with the most significant increase in phosphorylation level observed at the s445 site in PAAD compared to normal tissues (**Figure 7G**). Concurrently, in line with our findings shown in **Figure 1**, the abundance of MVP protein was markedly higher in PAAD than in normal tissues (**Figure 7H**).

Collectively, these results propose that the variations in promoter methylation level and protein phosphorylation status of MVP in PAAD may be a contributing factor to its tumorigenic potential.

### 3.8 Drug‐efficacy analysis and potential drug forecasting

**Figure 8:**
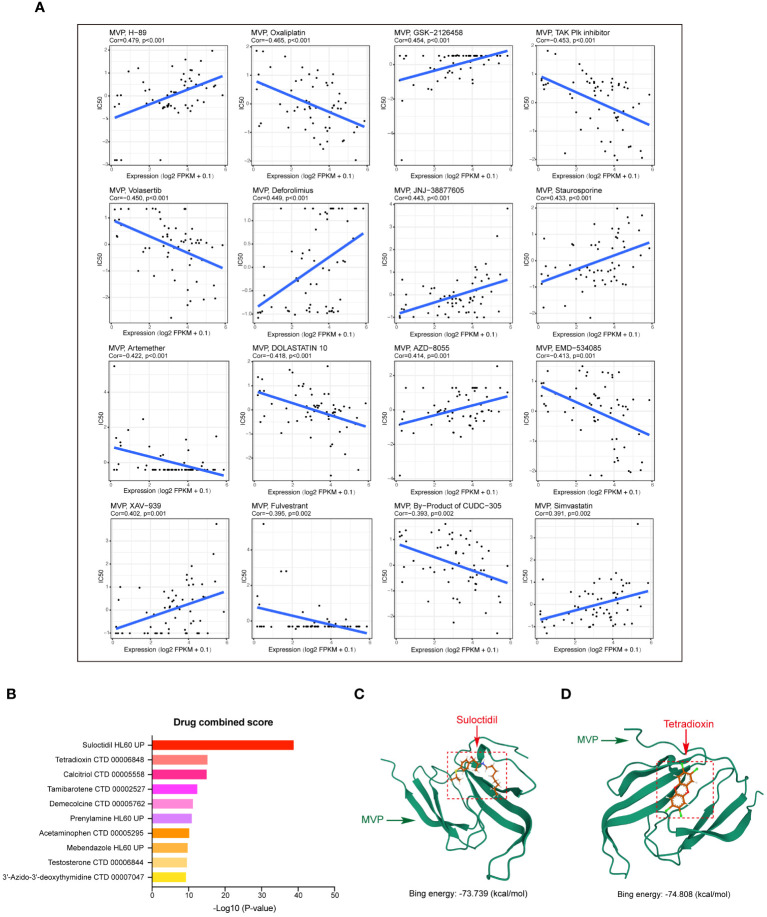
Drug‐efficacy analysis and potential drug prediction. **(A)** Scatter plots showing the correlations between MVP expression levels and the IC50 for a subset of most notable compounds from CellMiner. Gene expression data are normalized as (log2 FPKM+0.1). **(B)** Predicted drugs targeting MVP in PAAD from DSigDB. **(C, D)** The predicted binding modes of MVP with Sulodexide and Tetradioxin.

In this study, we explored the relationship between MVP expression in PAAD and the pharmacological efficacy of both (Food and Drug Administration) FDA-approved and clinically evaluated drugs using the data of RNA-seq and compound activity accessed from CellMiner. By calculating the Pearson correlation coefficient, we identified a significant association between the half-maximal inhibitory concentration (IC50) of a subset of compounds and MVP expression levels, the most notable of which were displayed in **Figure 8A**. Specifically, our analysis revealed a positive correlation between MVP expression levels and the IC50 values of several drugs, including H-89 (Cor=0.479), GSK-2126458 (Cor=0.454), Deforolimius (Cor=0.449), JNJ-38877605 (Cor=0.443), Staurosporine (Cor=0.433), AZD-8055 (Cor=0.414), XAV-939 (Cor=0.402) and Simvastatin (Cor=0.391). Conversely, we observed a negative correlation between MVP expression and the IC50 values of certain drugs like Oxaliplatin (Cor=-0.465), TAK Plk inhibitor (Cor=-0.453), and Volasertib (Cor=-0.450). These results suggest that MVP may serve as a biomarker for tailoring personalized therapeutic strategies for patients with PAAD, helping to optimize precision treatments for patients with elevated MVP levels.

Next, based on the molecular conformations of candidate compounds and their binding abilities with MVP, we further predicted small-molecular drugs targeting MVP for the treatment of PAAD. Utilizing the Drug Signatures Database (DSigDB) accessed through the Enrichr website, we identified a set of pharmacological compounds with specificity for MVP (**Figure 8B**). The chemical structural features of these compounds were subsequently obtained from PubChem, and they were prioritized according to their statistical significance (*P*-values) as illustrated in **Table 1**. Additionally, with AutoDock software, we forecasted the binding affinities and conformation of MVP with Suloctidil (**Figure 8C**) and Tetradioxin (**Figure 8D**), depicting their potential as MVP-targeted therapeutic drugs through the quantification of binding free energy and elucidation of interaction modalities.

**Table 1:** The chemical structural features of pharmacological compounds targeting MVP for the treatment of PAAD.

Term	P-value	Combined Score	Structure
Suloctidil HL60 UP	1.53E-39	6449.507	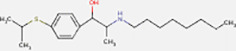
Tetradioxin CTD 00006848	6.84E-16	186.0601	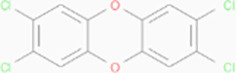
Calcitriol CTD 00005558	1.12E-15	215.1789	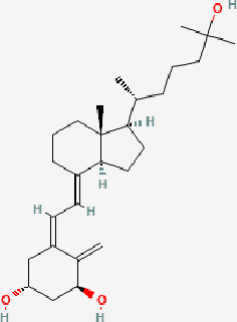
Tamibarotene CTD 00002527	4.27E-13	272.4829	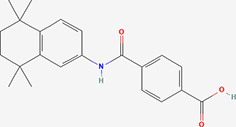
Demecolcine CTD 00005762	6.12E-12	182.2902	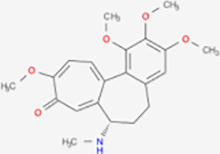
Prenylamine HL60 UP	1.27E-11	1445.056	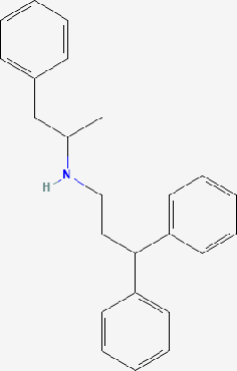
Acetaminophen CTD 00005295	6.55E-11	90.78105	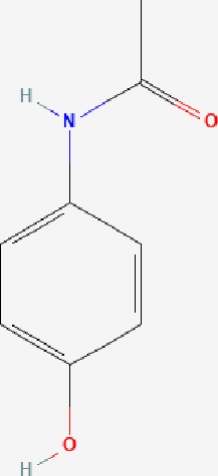
Mebendazole HL60 UP	1.84E-10	233.8491	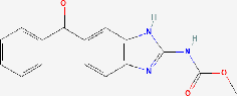
Testosterone CTD 00006844	2.87E-10	118.1044	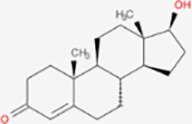
3’-Azido-3’-deoxythymidine CTD 00007047	5.42E-10	204.9508	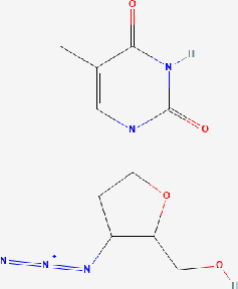

### 3.9 Antitumor effects of MVP knockdown in PAAD cell lines

Based on the multifaceted findings mentioned above, we have elucidated the oncogenic potential of MVP in driving tumorigenesis of PAAD and its feasibility as a biomarker for clinical diagnosis and prognosis of PAAD. This oncogene leads to a range of adverse prognostic effects by promoting cell proliferation and triggering immune dysfunction. To this end, we conducted a series of experiments to validate the potential inhibitory effects on malignant progression of tumors through targeted suppression of MVP at the cellular level.

**Figure 9:**
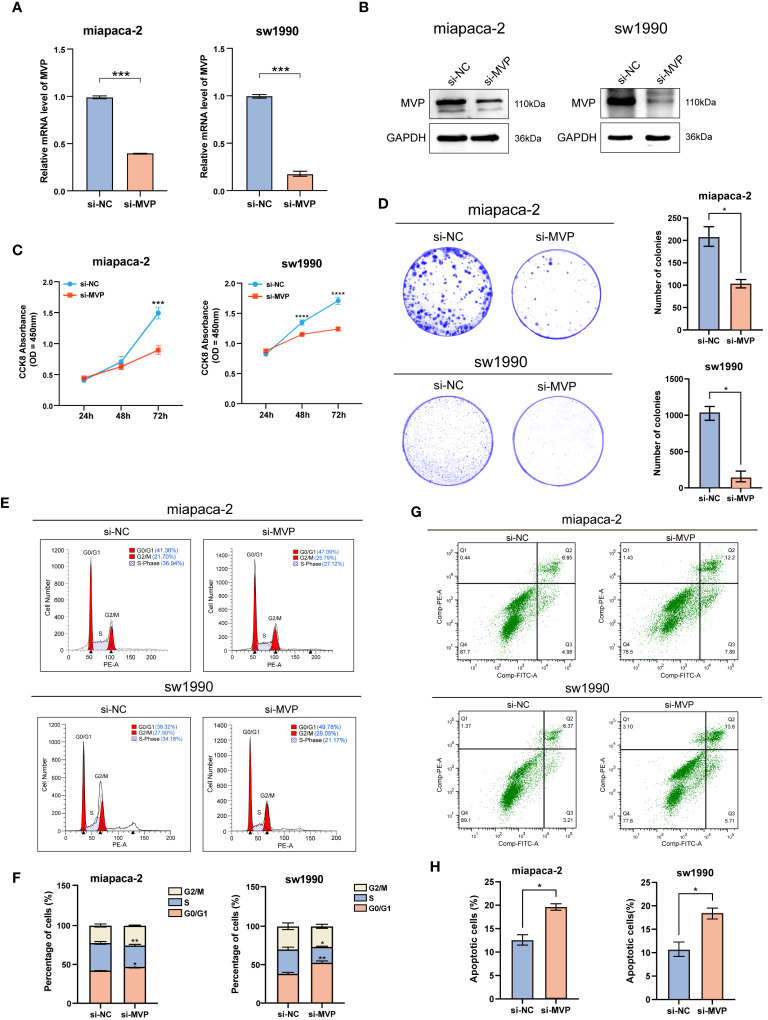
The effects of MVP knockdown on cell proliferation and apoptosis in PAAD cell lines. **(A)** Efficiency assessment of MVP knockdown at the mRNA level in PAAD cells. **(B)** Efficiency assessment of MVP knockdown at the protein level in PAAD cells. **(C)** The analysis of cell proliferation ability after MVP knockdown by CCK-8. **(D)** The colony formation assay after MVP knockdown. **(E, F)** The flow cytometry analysis of cell cycle distribution. **(G, H)** The flow cytometry analysis of cell apoptosis after MVP knockdown in PAAD cells. Statistical differences were assessed by using two-way ANOVA for **(C)**, and unpaired Student´s t-test for **(A, D, F, H)**, presented as the mean ± SD (*p < 0.05; **p < 0.01; ***p < 0.001; ****p < 0.0001).

At first, we verified the significant knockdown efficiency of siMVP in two PAAD cell lines (miapaca-2 and sw1990) at the transcriptional level and protein level via qRT-PCR and Western blot techniques, respectively (**Figures 9A, B**).

Next, Cell Counting Kit-8 (CCK-8) assay (**Figure 9C**) and colony formation assay (**Figure 9D**) indicated that knockdown of MVP inhibited the cell viability, showing significantly decreased proliferation of PAAD cell lines compared to negative controls (si-NC). The cell cycle was performed by flow cytometry analysis, which indicated a significant increase in the number of cells in the G0/G1 phase and a marked decrease in the number of cells in the S phase in the MVP knockdown (si-MVP) groups compared to the negative control (si-NC) groups, suggesting that blocking the expression of MVP in PAAD cell lines would partially hinder their proliferative capacity (**Figures 9E, F**).

Additionally, flow cytometry analysis detected elevated levels of apoptosis in PAAD cell lines following MVP downregulation (**Figure 9G**), with **Figure 9H** providing the quantified rates of total apoptosis for these cell lines.

**Figure 10:**
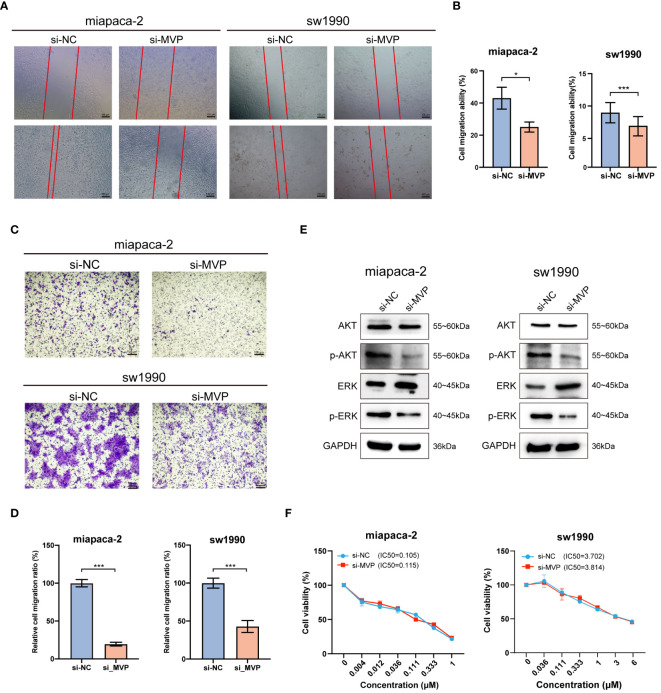
The effects of MVP knockdown on cell migration and related signaling pathway in PAAD cell lines. **(A, B)** Wound healing assay after MVP knockdown in PAAD cells. Representative images and quantitative analysis are shown, respectively. Scar bars, 100 μm. **(C, D)** The transwell assay to analyze cell migration ability. Representative images and quantitative analysis are shown, respectively. Scar bars, 100 μm. Statistical differences were assessed by using unpaired Student´s t-test, presented as the mean ± SD. (*p < 0.05; ***p < 0.001). **(E)** The protein expression level of ERK, p-ERK, AKT and p-AKT after MVP knockdown in PAAD cells by Western blot. **(F)** Cell viability assay for accessing the chemo-sensitivity of PAAD cells post-MVP knockdown. IC50: half-maximal inhibitory concentrations.

The migration ability of tumor cells is a fundamental element in the process of tumor metastasis. To explore the effects of MVP on the migration ability of PAAD cells, we performed wound healing assays and found that PAAD cells in the absence of MVP exhibited a severe decline in the migratory capacity (**Figures 10A, B**). This finding was corroborated by transwell assays, which also demonstrated a significant impairment in migrative ability of PAAD cells upon MVP knockdown (**Figures 10C, D**).

Previous studies have reported that MVP suppresses the progression of papillary thyroid cancer by interfering with the ERK and AKT pathways. Hence, we wondered whether MVP exerts a similar inhibitory effect on these pathways in PAAD. In this regard, through Western blot analysis, we observed a decrease in the expression levels of phosphorylated ERK (p-ERK) and phosphorylated AKT (p-AKT) proteins after MVP knockdown (**Figure 10E**).

Moreover, we performed the cell viability assay to evaluate the chemo-sensitivity of PAAD cells following MVP knockdown. The results indicated that the IC50 values were comparable to those of the negative controls (si-NC), suggesting no significant change in chemo-resistance to Gemcitabine, the standard chemotherapeutic agent (**Figure 10F**).

In summary, knockdown of MVP in PAAD cell lines impairs cell proliferation and migration abilities, accelerates cell apoptosis, and thereby inhibits the tumor progression, which strongly supports the results of our bioinformatics analyses.

## 4 Discussion

Globally, PAAD is one of the most aggressive cancers and a major public health challenge that necessitates further research into effective biomarkers and therapeutic targets (23). In this study, we first comprehensively elucidated the role of MVP in PAAD. We conducted the first-ever analysis of the expression pattern of MVP in a large cohort containing 370 PAAD patients from TCGA and GEO datasets, which consistently demonstrated a markedly increased expression of MVP in PAAD tissues. Importantly, this elevated expression was found to be significantly correlated with an adverse prognosis in PAAD patients. A series of related analyses further confirmed its potential as a novel diagnostic and prognostic indicator for PAAD patients, which is also consistent with reports in a variety of other cancer contexts (7–11).

To gain a nuanced understanding of the factors contributing to the abnormal expression of MVP in PAAD, we explored the characteristics of MVP-associated genomic alterations, promoter methylation, and phosphorylation. Notably, we identified genetic alterations in 1.62% of PAAD samples, coupled with a lower level of promoter methylation compared to normal tissues. Given that previous studies have associated aberrant methylation with an increased risk of PAAD development (24) and that promoter hypomethylation of oncogenes has been implicated as a promotive factor of tumorigenesis (25, 26), our observations suggest that the abnormal hypomethylation status of MVP may play a potential role in the initiation and progression of PAAD, which would result in gene overexpression, and activation of downstream signaling pathways. Additionally, phosphorylation stands out as one of the most prevalent and crucial post-translational modifications (PTM). Mutations occur at protein phosphorylation sites can trigger the initiation and progression of malignant tumors (27). Our results indicated a notable increase in both protein abundance and protein phosphorylation of MVP gene at the s445 site in PAAD samples compared to normal tissues, suggesting its potential involvement in the malignant development of the tumor. Taken together, genetic alterations, aberrant methylation and phosphorylation levels of MVP may contribute to the development of PAAD.

Moreover, via the STRING database, we uncovered proteins that potentially interact with MVP, such as PTEN and PARP1, which occupy central positions in the protein-protein interaction network. PTEN has been reported to possess nuclear localization signal-like sequences required for MVP-mediated nuclear translocation (28). PARP1 is a key DNA nick sensor, and inhibitors of PARP1 have exhibited promising results in clinical trials for PAAD (29). In addition, we investigated the possible downstream mechanisms by which MVP plays an oncogenic role in PAAD using GO, KEGG and GSEA enrichment analyses. GO enrichment analysis suggested that MVP was associated with digestion and O-glycan processing, KEGG pathways was enriched in signaling pathways such as pancreatic secretion, and GSEA enrichment analysis indicated a connection between MVP and immune-related processes in PAAD.

To unravel the connection mentioned above, we conducted a comprehensive investigation into various aspects of the TME, where immune regulation drives cell plasticity and drug response (30). TIICs play a critical role in the TME, influencing both tumor progression and immunotherapy responses (31). We revealed the subtle dynamic interactions between MVP and TME by delving into the intricate relationship between MVP and TIICs. Utilizing data from Timer2.0, we discovered a significant association between MVP expression levels and a cohort of TIICs, and of particular note, Th17 cells may tend to promote tumor cell proliferation by releasing interleukin-17 (IL-17) (32), which imply that MVP might facilitate tumorigenesis and aggravation in PAAD.

Besides, we explored the relationship between MVP expression and ICI therapy based on TIDE scores. Our analysis revealed that a decrease in MVP expression indicated an enhanced tendency for PAAD patients to benefit from ICI therapy. This aligns with the better prognostic outcomes observed in the MVP low-expression group, highlighting the potential of MVP as a predictive biomarker for ICI responsiveness in PAAD. Further analysis of immune-related factors from the TISIDB website depicted a negative correlation between MVP expression and immunostimulatory factors, coupled with positive correlations with immunoinhibitors, immune checkpoints, chemokines, and chemokine receptors.

T cells constitute a crucial subset within the diverse subpopulation of TIICs and exhibit a preference for tissue distribution and dynamic properties (33). Traditional methods have limitations in capturing the intricate diversity of T cell populations and the advent of emerging single-cell technologies provides a powerful avenue for dissecting and comprehending the distribution of distinct T cell subsets within TIICs. Thus, we investigated the intricate relationship between MVP expression levels and the dynamic distribution of immune cells at the single-cell level through the TISCH website, showing a higher proportion of malignant cells in tumor tissues than that in normal tissues. UMAP plots revealed a widespread distribution of MVP across various immune cell types, with the highest percentage observed in malignant cells. The prevalence of MVP in malignant cells suggests a potential role in shaping the immune microenvironment of PAAD, highlighting the urgent need for future studies to dissect the molecular mechanisms underlying this interaction.

Additional analysis on drug prediction were based on RNA-seq and compound activity data from CellMiner, which showed a significant correlation between MVP expression levels and the efficacy of specific compounds. Notably, the observation of a negative correlation between MVP expression and the IC50 of oxaliplatin, a platinum-based anticancer drug currently serving as the standard adjuvant therapy for resection of PAAD (34), holds significant clinical implications. This intriguing finding implies a potential avenue for optimizing therapeutic strategies by tailoring oxaliplatin administration for patients with elevated MVP expression in PAAD. This pharmacological correlation suggests that MVP expression could serve as a biomarker for tailoring therapeutic strategies in PAAD, guiding the precision treatments based on the MVP levels in individual patients. In light of these findings, we propose MVP as a pivotal therapeutic target within the context of PAAD management. Upon drug databases and structural analysis, we further identified Suloctidil and Tetradioxin as potential MVP-specific targeted pharmacological agents with binding affinities and configurations that warrant further exploration.

To validate the results of the bioinformatics analysis, we detected the effects of MVP deficiency on multiple biological processes, such as cell proliferation, apoptosis, viability, and migration, through a series of cellular experiments, thus providing functional insights into the impacts of MVP on PAAD cells. Our findings clarified that MVP knockdown considerably impeded cell proliferation, induced shifts in cell cycle dynamics, elicited apoptotic responses, and attenuated cell migration. These findings elucidate the important role of MVP in driving key hallmarks of cancer and substantiate its potential as a therapeutic target.

Overall, this study systematically analyzed the multifaceted roles of MVP in the complex immune microenvironment of PAAD based on a multi-omics analysis of clinical samples and validation experiments. We clarified that MVP promoted the proliferation and migration of PAAD cells and had the potential to serve as an immune-related biomarker of PAAD, opening avenues for targeted therapies and personalized treatment strategies. Our findings will not only contribute to further elucidating of the regulatory mechanisms of MVP in the progression and treatment of PAAD, but also provide new insights for developing new drugs suitable for PAAD patients and optimizing personalized therapeutic plans.

## 5 Conclusions

In summary, this study shed light on the role and regulatory mechanism of MVP in PAAD, primarily focusing on analyzing the relationship between the expression of MVP and tumorigenesis, immune infiltration and immunotherapy. Additionally, the DNA methylation and phosphorylation status of MVP has been detected, and the effective small molecule drugs that targeted MVP for the treatment of PAAD has been predicted. Our findings elucidate that MVP is closely associated with TME and promoting the occurrence and development of PAAD, indicating its potential as a novel and reliable clinical diagnostic and prognostic indicator. This study not only comprehensively reveals the role of MVP in PAAD and its potential as a promising immune-related biomarker, but also opens new avenues for targeted and personalized treatment modalities.

## Data availability statement

The original contributions presented in the study are included in the article/**Supplementary Material**. Further inquiries can be directed to the corresponding author.

## Ethics statement

Ethical approval was not required for the studies on humans in accordance with the local legislation and institutional requirements because only commercially available established cell lines were used.

## Author contributions

XW: Conceptualization, Data curation, Formal analysis, Investigation, Methodology, Validation, Writing – original draft, Visualization. LH: Formal analysis, Investigation, Validation, Writing – original draft. JL: Data curation, Formal analysis, Software, Writing – original draft. XG: Methodology, Validation, Writing – original draft. YL: Project administration, Resources, Supervision, Writing – review & editing. CL: Conceptualization, Data curation, Formal analysis, Investigation, Project administration, Supervision, Validation, Visualization, Writing – review & editing, Funding acquisition.
