# Supplementary figures and images for: Unraveling the role of Major Vault Protein as a novel immune-related biomarker that promotes the proliferation and migration in pancreatic adenocarcinoma
Source: Front Immunol. 2024 Jul 4;15:1399222. doi: 10.3389/fimmu.2024.1399222 (PMC11254802; doi:10.3389/fimmu.2024.1399222)

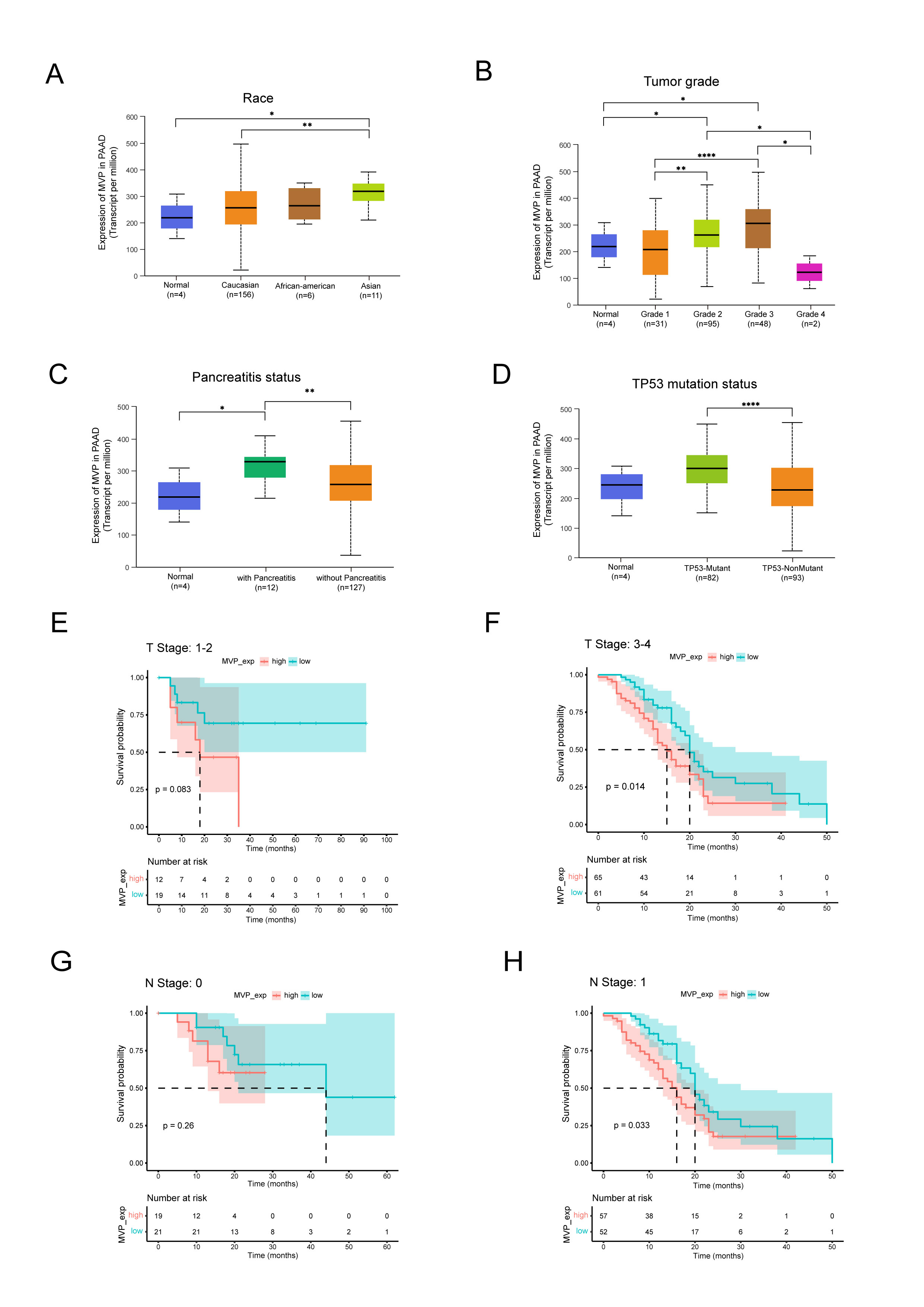

Supplement: Supplementary file 1 [file Image_1.jpeg]

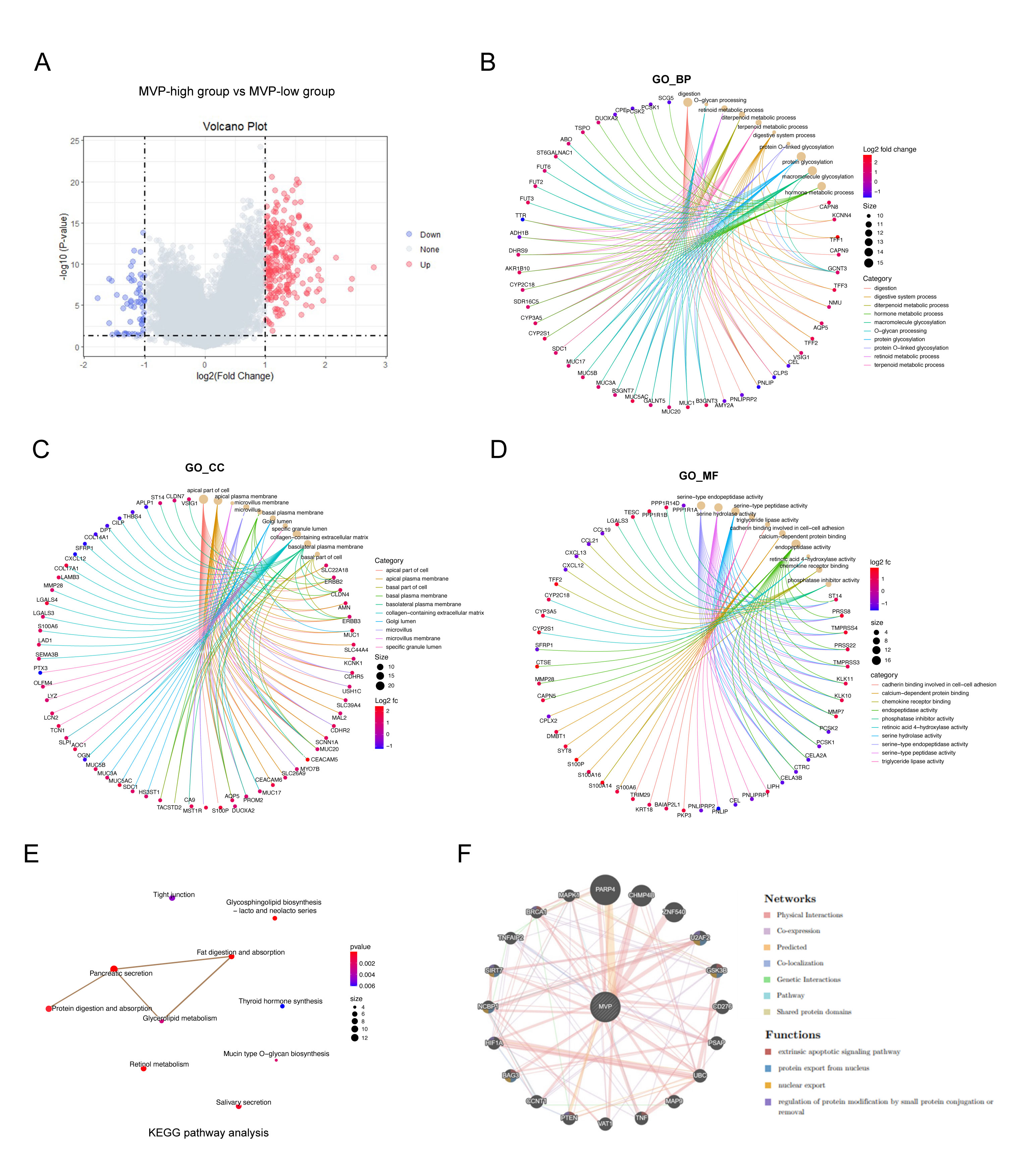

Supplement: Supplementary file 2 [file Image_2.jpeg]

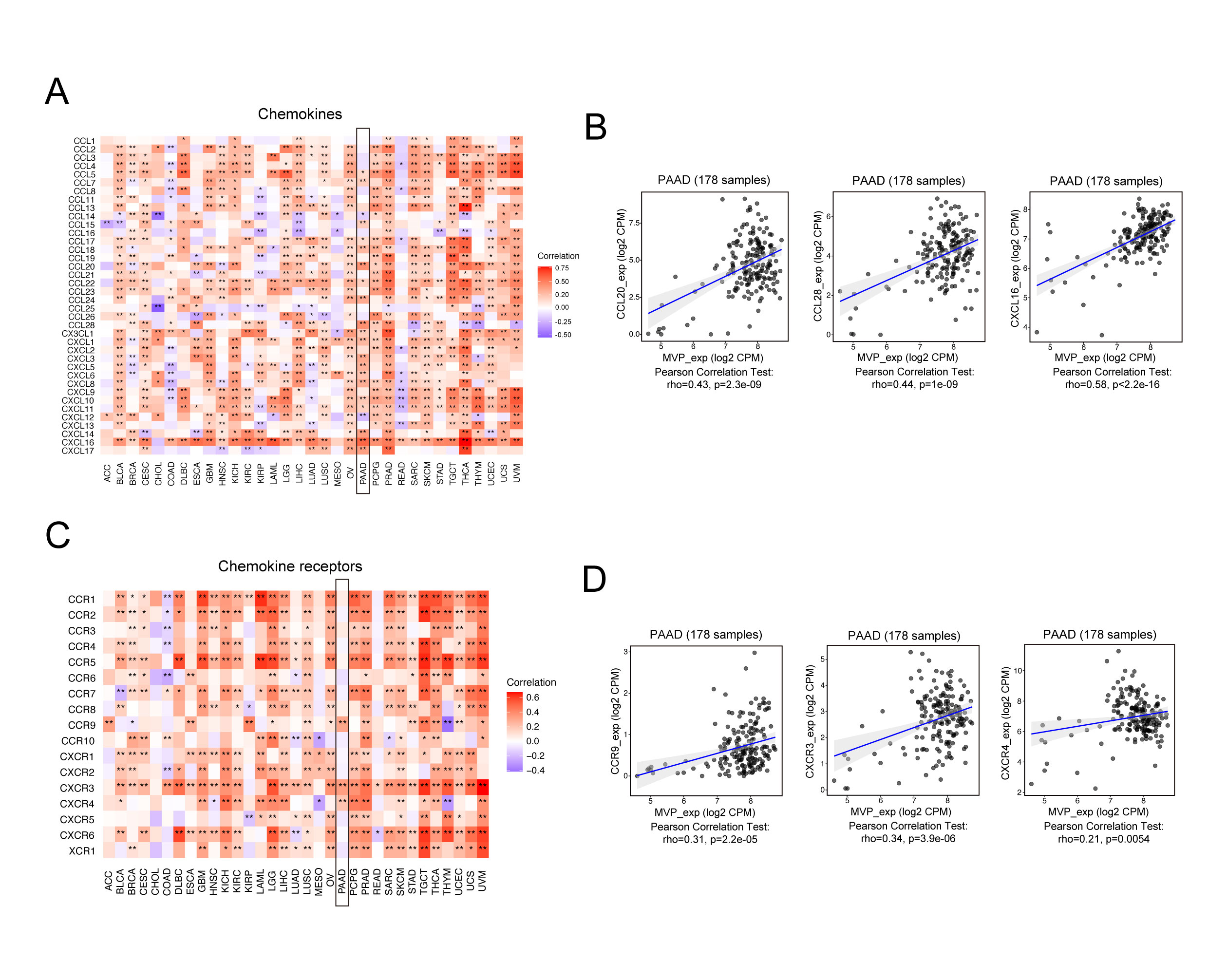

Supplement: Supplementary file 3 [file Image_3.jpeg]
